# Work Environment Surrounding COVID-19 Outbreak in Call Center, South Korea

**DOI:** 10.3201/eid2610.202647

**Published:** 2020-10

**Authors:** Taeshik Kim

**Affiliations:** Seoul Metropolitan Government–Seoul National University Boramae Medical Center, Seoul, South Korea

**Keywords:** COVID-19, 2019 novel coronavirus disease, SARS-CoV-2, severe acute respiratory syndrome coronavirus 2, viruses, respiratory infections, zoonoses, work environment, workplace, presenteeism, sick leave, sick benefits, occupational illness

**To the Editor:** I read with interest the recent synopsis by Park et al. (*1*) about a coronavirus disease outbreak in a call center, in which I was involved as a field epidemiologist. I would like to share my perspective as an occupational physician.

The work environment of the call center was an important reason for the high attack rate on the 11th floor. The width of the desks was 1.2 m, and most employees had worked without face masks despite the high risk for severe acute respiratory syndrome coronavirus 2 transmission associated with having persons continuously engaged in phone calls through headsets in an enclosed space. Call centers are known for their poor working conditions, the lack of power among employees, and high demands of the job (https://www.diva-portal.org/smash/get/diva2:20713/fulltext01.pdf).

In addition, presenteeism (i.e., attending work while ill) also affected the high attack rate (*2*,*3*). At least 10 employees continued to work despite having symptoms. In South Korea, sick leave and other benefits are not available for most workers (*4*). Given the lack of sick leave and concerns about disincentives for absences, employees could not have left the workplace easily. Without sick leave, workers are reluctant to apply for workers’ compensation, the only alternative, and employers avoid registering workplace accidents for fear of penalties. These factors explain why the occupational accident rate does not reflect reality. A paradoxical discrepancy has been observed between South Korea and the average European Union country in both lower occupational accident rates (484 vs. 1,558/100,000 workers) and higher fatal accident rates (10.54 vs. 1.65/100,000 workers) (*5*).

The outbreak in the call center reflects the work environment and compensation system in South Korea. To prevent transmission of severe acute respiratory syndrome coronavirus 2 in the workplace, South Korea needs not only improvements in physical working conditions (e.g., use of physical distancing and telework) but also introduction of sick leave and a more accessible workers’ compensation system.
